# Case Report: A case of interstitial pneumonia in a colorectal cancer patient with a history of lung cancer treated with cetuximab

**DOI:** 10.3389/fonc.2025.1541649

**Published:** 2025-07-24

**Authors:** Yang Yang, Bin Zhang, Hongjun Li, Mei Ren, Daoqun Chong, Yujin Guo, Haibo Zhao, Qing-Qing Yu

**Affiliations:** ^1^ Department of Oncology, Jining No.1 People’s Hospital, Jining, China; ^2^ School of Pharmaceutical Engineering, Jining Medical University, Jining, China; ^3^ Clinical Pharmacology, Jining No.1 People’s Hospital, Jining, China; ^4^ Clinical Research Center, Jining No.1 People’s Hospital, Jining, China

**Keywords:** cetuximab, interstitial lung disease, colorectal cancer, personalized therapy, EGFR inhibitors

## Abstract

**Purpose:**

This study aims to investigate the pathogenesis, clinical manifestations, and prognosis of interstitial lung disease (ILD) induced by cetuximab.

**Patients and methods:**

A single case of a colorectal cancer patient who underwent radical resection and descending colon-rectal anastomosis for sigmoid colon cancer on October 22, 2020, was selected. Postoperative pathology revealed moderately differentiated adenocarcinoma confined to the mucosa, measuring approximately 0.5×0.2×0.2 cm, with no cancer involvement in the proximal and distal resection margins, and metastatic carcinoma observed in 1 out of 5 pericolic lymph nodes. On July 29, 2021, due to a significant increase in CA19–9 levels, chest and abdominal CT scans, along with brain MRI, indicated enlarged retroperitoneal lymph nodes. The patient was afebrile and without respiratory symptoms such as cough, sputum production, chest tightness, or shortness of breath, and imaging suggested disease progression. With no significant abnormalities in C-reactive protein, blood routine, and no evidence of infection, cetuximab-related ILD was considered. Due to mild inflammation, the patient continued cetuximab combined with chemotherapy.

**Results:**

This study suggests that cetuximab can induce ILD, and the onset time may be associated with factors such as serum albumin levels and underlying pulmonary diseases.

**Conclusion:**

The incidence of cetuximab-induced ILD is low and relatively rare, with non-specific clinical symptoms. Clinically, treatment plans should be tailored to the patient’s actual condition to control disease progression and improve patient prognosis.

## Introduction

Cetuximab, a chimeric monoclonal antibody targeting the epidermal growth factor receptor (EGFR), is generated by combining the constant region of human immunoglobulin with the antigen-binding region of a mouse antibody. It is widely used in combination with chemotherapy to treat advanced colorectal cancer (CRC), where it has demonstrated favorable therapeutic outcomes (1, 2). CRC, one of the three most prevalent cancers worldwide, accounts for approximately 10% of cancer cases and ranks as the second leading cause of cancer-related mortality globally, following lung cancer (3). As a recombinant chimeric immunoglobulin G1 (IgG1) monoclonal antibody and EGFR inhibitor, cetuximab inhibits EGFR overexpression in cancer cells. This overexpression contributes to tumor progression by activating cell proliferation, inhibiting apoptosis, enhancing cell motility, and promoting metastasis and invasion (4).

**Figure 1:**
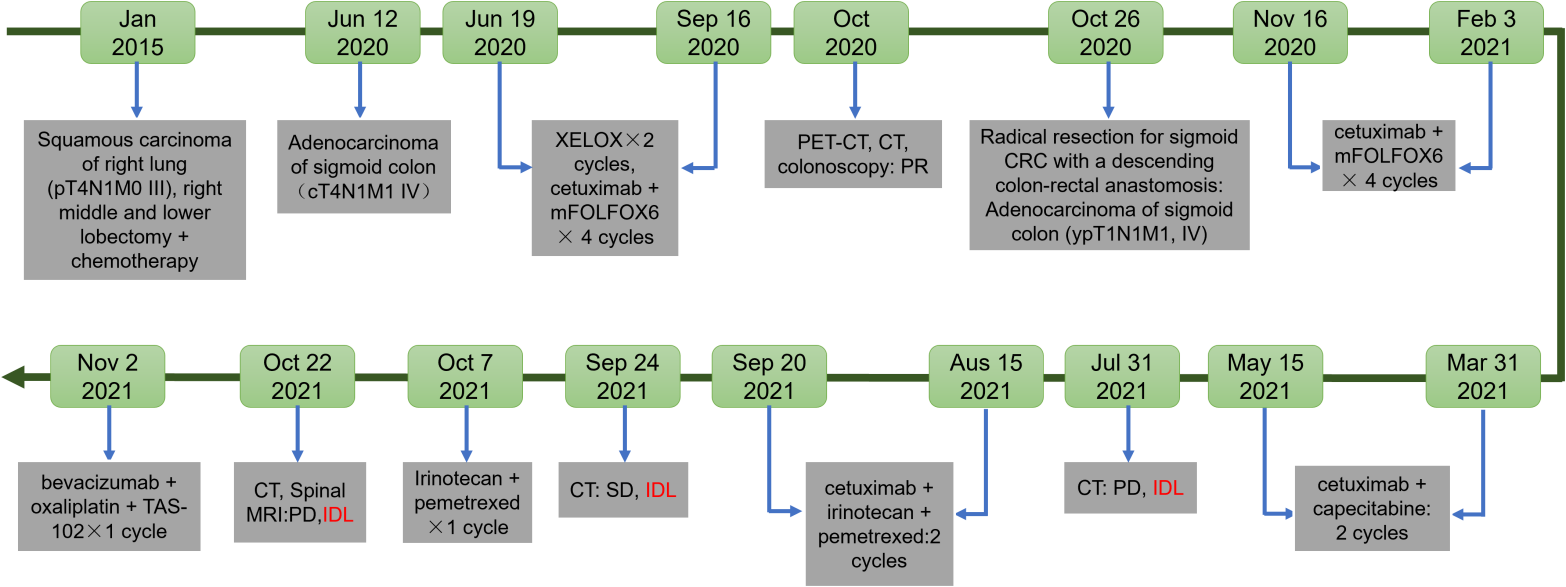
Timeline of the clinical course.

Skin toxicity is the most frequently reported adverse effect associated with cetuximab treatment (5). Other side effects include fatigue, headaches, gastrointestinal disturbances, and infusion reactions, with lung injury being an uncommon occurrence (6). Cetuximab-induced interstitial lung disease (ILD) has been reported in patients with various malignancies, including head and neck cancer, bladder cancer, CRC, and lung cancer (7, 8). This report presents a case of cetuximab-induced ILD in a patient with CRC and a prior history of lung cancer, emphasizing the importance of recognizing and addressing potential pulmonary complications in patients undergoing cetuximab therapy (**Figure 1**).

## Clinical information

A male patient was presented to the thoracic surgery department of Jining NO.1 People’s Hospital on January 20, 2015, with a right lung mass. Following a comprehensive diagnostic evaluation, the patient underwent a right middle and lower lobectomy with mediastinal lymph node dissection on January 30, 2015. Postoperative pathological analysis revealed moderately differentiated squamous cell carcinoma of the right middle and lower lobes. The tumor measured 5.5 × 3.5 × 3 cm and showed pleural invasion. Metastasis was observed in one of five examined peribronchial lymph nodes. The patient subsequently completed four cycles of gemcitabine and cisplatin chemotherapy. Cytological examination of right-sided pleural effusion revealed no atypical cells, and testing for EGFR mutations was negative.

On June 3, 2020, routine physical examination and computed tomography (CT) imaging revealed slightly enlarged retroperitoneal lymph nodes of indeterminate nature, raising suspicion of metastatic disease. Magnetic resonance imaging (MRI) of the upper abdomen confirmed multiple enlarged retroperitoneal lymph nodes. At the same time, cervical lymph node ultrasound identified multiple hypoechoic nodules in the left supraclavicular fossa. On June 9, fine needle aspiration of the cervical lymph nodes confirmed metastatic carcinoma, though adenocarcinoma could not be excluded. The genetic analysis showed that EGFR, KRAS, BRAF, PIK3CA, NRAS, HER2, MET, AKT1, c-KIT, ALK, PDGFRA, RET and ROS1 were negative. PD-L1 tumor proportion score (TPS) was <1%. Tumor marker analysis revealed elevated levels of carcinoembryonic antigen (CEA) at 39.19 ng/mL, Carbohydrate antigen (CA) 19–9 at 346.5 U/mL, CA72–4 at 21.76 U/mL, and Cyfra211 at 4.9 ng/mL

On June 12, a colonoscopy revealed an adenomatous polyp in the ascending colon, which was biopsied. A 2.0 cm polyp with surface depression and mucosal erosion was identified in the sigmoid colon, from which four biopsy samples were obtained. The remaining segments of the ascending, transverse, descending colon, sigmoid colon, and rectum were partially covered with fecal matter and showed several hemispherical polyps (0.5–0.8 cm in diameter) with smooth mucosa, normal coloration and clear vascular patterns and no signs of ulceration or masses. Pathological examination confirmed adenocarcinoma within the mucosal tissue of the sigmoid colon, while tissue from the ascending colon showed tubular adenoma changes.

Based on these findings, the patient was diagnosed with sigmoid colon adenocarcinoma with metastasis to the left supraclavicular and retroperitoneal lymph nodes. Systemic chemotherapy with XELOX (oxaliplatin 230 mg on day 1 and capecitabine 2.0 g qds for 14 days) was initiated for two cycles on June 19, 2020, resulting in disease stabilization. Then, the patient underwent four cycles of cetuximab combined with mFOLFOX6 (cetuximab 800 mg on day 0, oxaliplatin 150 mg on day 1, leucovorin 0.75 g on day 1, and fluorouracil 0.75 g on day 1, with continuous intravenous infusion for 46 hours) on July 31, August 20, September 3, and September 16, 2020.

On October 13, a positron emission tomography-computed tomography (PET-CT) scan revealed no significant thickening of the sigmoid colon wall or abnormal metabolic activity. There were no signs of increased metabolic activity in the postoperative region of the right lung. Linear opacities in both lungs were consistent with chronic inflammation. On October 14, PET-CT imaging confirmed postoperative changes in the right lung cancer site, showing multiple linear foci and small nodules in the right lung, right pleural effusion, thickened and adherent right pleura, and slight wall thickening in the sigmoid colon. PET-CT findings showed no evidence of metastasis or primary lesions. A follow-up colonoscopy identified multiple colon polyps, which underwent high-frequency electrocautery and ablation, including marked lesions in the sigmoid colon. Biopsy pathology indicated tubular adenomas in the ascending and transverse colon.

On October 22, 2020, the patient underwent radical resection for sigmoid CRC with a descending colon-rectal anastomosis. Postoperative pathological examination revealed moderately differentiated adenocarcinoma confined to the mucosa. The tumor measured approximately 0.5 × 0.2 × 0.2 cm under microscopic evaluation. The proximal and distal resection margins were free of cancer invasion, while metastatic carcinoma was observed in 1 out of 5 pericolic lymph nodes. Immunohistochemical analysis showed the following profile: MLH1 (+), weakly positive for PMS2 (weak +), MSH2 (+), MSH6 (+), CK7 (-), CK20 (+), CDX-2 (+), Catenin-β (+), SATB2 (+), PSA (-), and focally positive for P504S (local +). The genetic analysis showed that no mutation in KRAS, NRAS, BRAF, NTRK1/2, ERBB2 (HER2).and microsatellite instability (MSI) testing confirmed stability. Based on these findings, the cancer was classified as sigmoid colon adenocarcinoma, ypT1N1M1, Stage IV. The patient reported no symptoms of cough, chest tightness, or dyspnea during the postoperative period.

Postoperative follow-up included 4 cycles of systemic chemotherapy with cetuximab (800 mg) combined with mFOLFOX6. This regimen, administered on November 16, December 8, December 27, 2020, and January 13, 2021, included cetuximab (800 mg on day 0), oxaliplatin (150 mg on day 1), leucovorin (0.75 mg on day 1), and fluorouracil (0.75 g on day 1, infused continuously over 46 hours). In March 2021, following four cycles of chemotherapy, an electrocardiogram (ECG) revealed an abnormal Q wave in leads II, III, and aVF. Coronary CT identified calcified and non-calcified plaques in the left anterior descending artery, causing mild luminal stenosis. Despite these findings, the patient denied symptoms of chest pain, tightness, shortness of breath, or precordial discomfort. A cardiology consultation led to a diagnosis of unstable angina associated with coronary atherosclerotic heart disease. From March 31 to May 15, 2021, the patient received maintenance chemotherapy, receiving cetuximab (800 mg every two weeks) combined with capecitabine (1.5 g twice daily on days 1–14 every three weeks).

**Figure 2:**
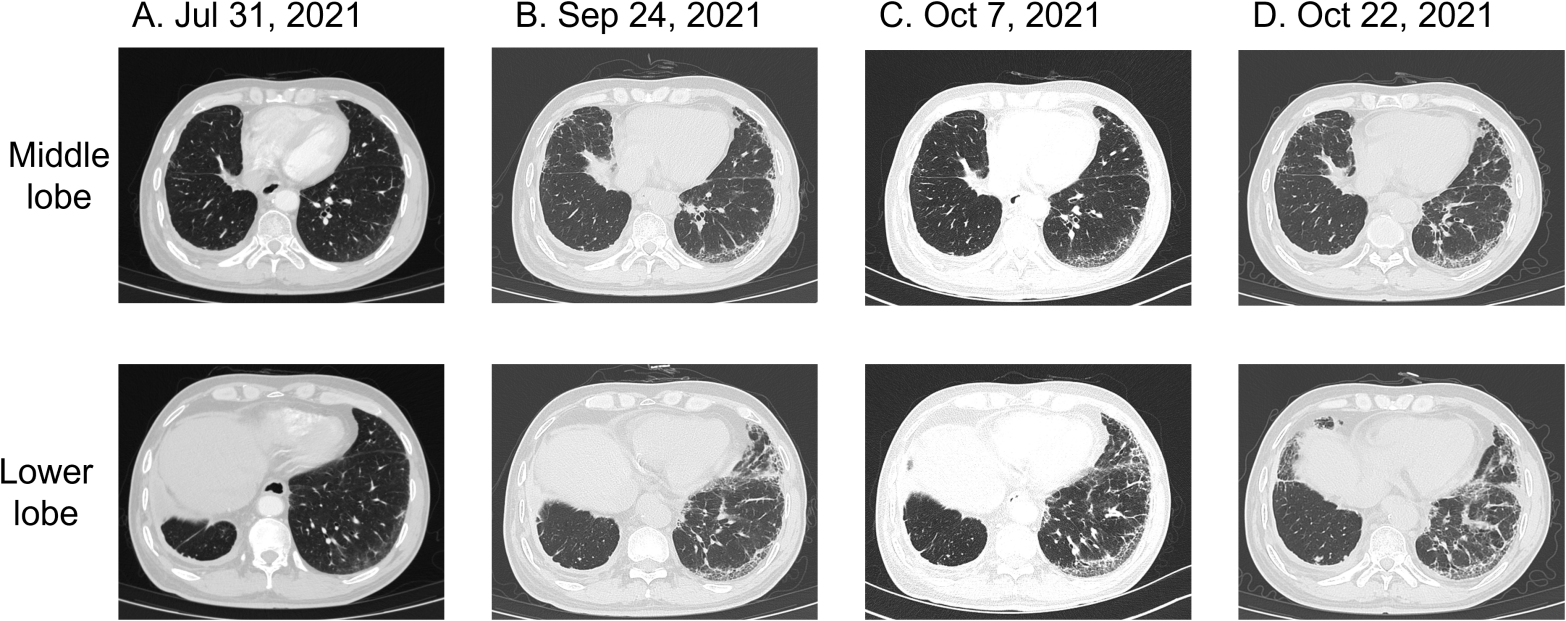
Pulmonary CT findings of interstitial pneumonia associated with cetuximab treatment for colorectal cancer. **(A)** Chest CT on July 31, 2021: mild interstitial changes in both lungs. **(B)** Chest CT on September 24, 2021: bilateral interstitial inflammation. **(C)** Chest CT on October 7, 2021: persistent interstitial fibrosis in both lungs. **(D)** Chest CT on October 22, 2021: mild progression of interstitial inflammation and localized fibrosis in both lungs, accompanied by pulmonary emphysema.

On July 29, 2021, the patient showed a significant elevation in CA19–9 levels, which reached 108 U/mL Chest and abdominal CT scans, along with a brain MRI performed on July 31, revealed retroperitoneal lymphadenopathy, mild interstitial changes in both lungs, and signs of disease progression. Despite these findings, the patient remained asymptomatic for fever, cough, sputum production, chest tightness, or shortness of breath (**Figure 2A**). Routine laboratory tests, including C-reactive protein and complete blood count showed no abnormalities. Physical examination identified slightly decreased breath sounds in the right lung and coarse breath sounds in the left lung, without evidence of dry or moist rales. Starting on August 15, 2021, the patient received a regimen of cetuximab (800 mg on day 0), irinotecan (320 mg), and pemetrexed (3.6 mg) every two weeks for three cycles. After completing three cycles, on September 20, 2021, the patient developed a mild cough with occasional white sputum but reported no fever, dyspnea, or chest tightness. Physical examination revealed decreased breath sounds in the right lung and coarse breath sounds in the left lung, without dry or moist rales. A follow-up CT scan conducted on September 24, 2021, revealed postoperative changes in the right lung, chronic bronchitis, pulmonary emphysema, bilateral interstitial inflammation, multiple small pulmonary nodules, a small right-sided pleural effusion, and thickening of the right pleura. Fibrotic strands were observed in both lungs, particularly in the peripheral regions, indicating the progression of interstitial changes compared to the previous imaging (**Figure 2B**). Laboratory tests showed serum albumin at 39.7 g/L (reference ranges:40-55g/L), an elevated C-reactive protein level of 8.47 mg/L, and an erythrocyte sedimentation rate of 23 mm/h, while complete blood count, rheumatoid factor, and antinuclear antibody profiles showed no significant abnormalities. A consultation with the respiratory department on September 30 raised concerns about exacerbated cetuximab-induced interstitial pneumonia with consultations as follows: “note to review the chest CT within one month and complete the following tests: erythrocyte sedimentation rate (ESR), antinuclear antibody panel (ANA), two rheumatology-related tests, and anti-neutrophil cytoplasmic antibody (ANCA) test (29 September, 2021)”; “it is recommended to discontinue the medication for observation. Currently, the patient has no obvious symptoms of shortness of breath, and hormone therapy can be temporarily not initiated (30 September, 2021)”. This raises the possibility of cetuximab-associated ILD as a potential contributing factor to the observed changes. The following alternative etiologies were systematically evaluated: Progression of pre-existing lung disease was considered improbable given the patient’s prior lung cancer was in remission with no radiographic evidence of recurrence. Infection was excluded based on negative serial cultures, PCR for pneumocystis, and viral panels. Among chemotherapeutic agents, although pemetrexed and irinotecan are known to potentially induce ILD, the temporal correlation of radiographic changes most strongly aligns with cetuximab administration. Given the relatively mild inflammatory presentation, cetuximab therapy was continued in conjunction with chemotherapy. The decision to proceed with cetuximab was guided by several key considerations: the presence of only mild radiographic changes without accompanying clinical symptoms (e.g., dyspnea or hypoxia), a multidisciplinary assessment weighing the risks of interstitial lung disease (ILD) progression against the benefits of ongoing cancer control, and vigilant monitoring via serial imaging studies. It is important to note that the patient was thoroughly informed of the potential risks and provided informed consent for the continuation of therapy. Therefore, cetuximab was discontinued. As the patient did not show dyspnea, corticosteroid therapy was deferred, and pulmonary symptoms were closely monitored. Due to elevated liver enzymes (ALT 84.2 U/L, AST 95.0 U/L) indicating liver impairment, only supportive liver therapy was provided.

**Figure 3:**
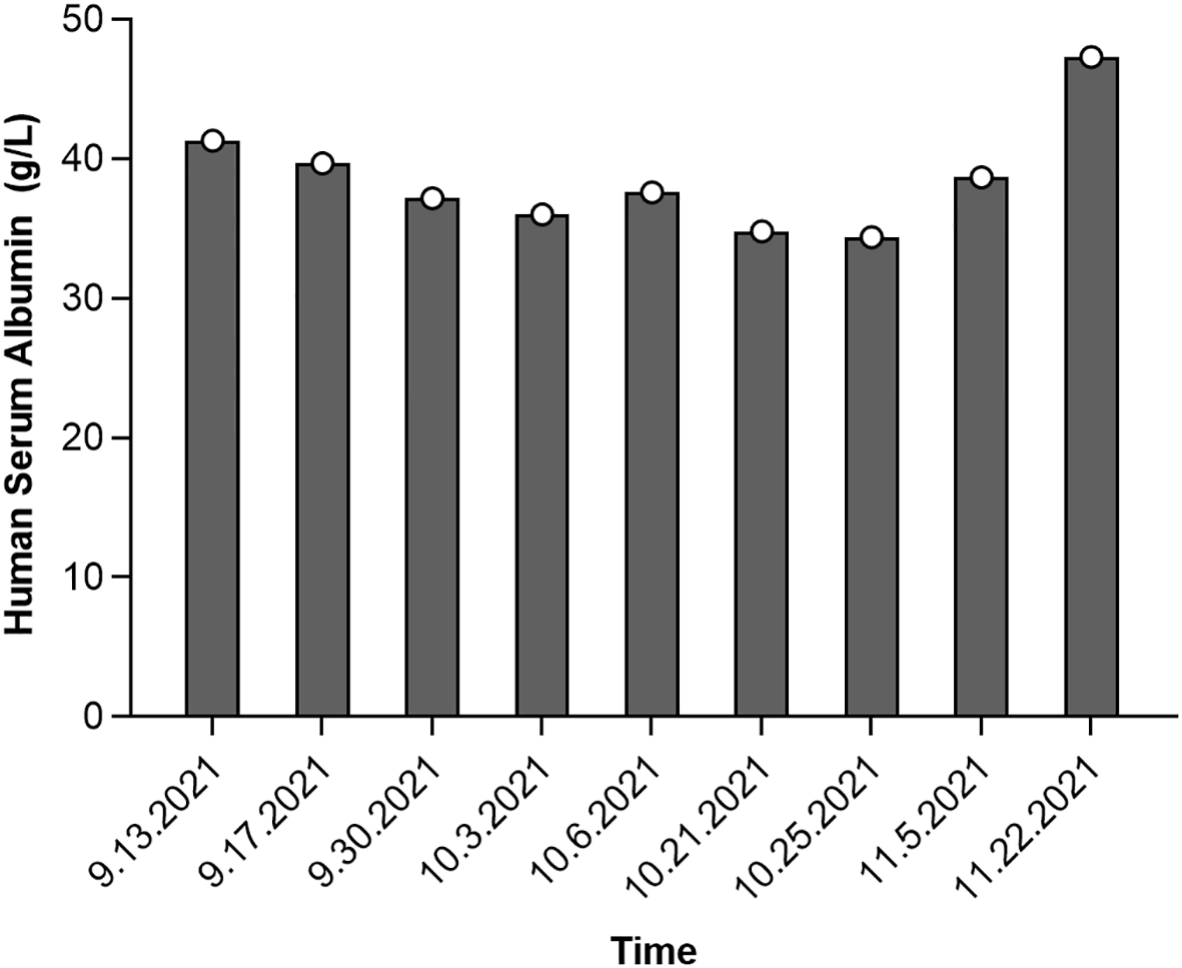
Serum albumin level changes during the development of interstitial pneumonia following cetuximab treatment for colorectal cancer.

Follow-up biochemical tests on October 3, 2021, revealed serum albumin at 36 g/L, subsequently at 37.6 g/L on October 6, 2021. A repeat chest CT on October 7, 2021, showed persistent interstitial fibrosis in both lungs, multiple small nodular foci, scattered patchy opacities, and a small right-sided pleural effusion and localized pleural thickening. These findings were consistent with the previous CT scan on September 24 (**Figure 2C**). Chemotherapy with irinotecan (320 mg on day 1) and pemetrexed (3.6 mg on day 1) was initiated on October 7, 2021. Despite treatment, serum albumin levels decreased to 34.8 g/L by October 21, with further reduction to 34.4 g/L by October 25. However, by November 5, serum albumin levels improved to 38.7 g/L, and its continuous changes are presented in **Figure 3**. A CT scan on October 22 revealed mild progression of interstitial inflammation and localized fibrosis in both lungs, accompanied by pulmonary emphysema, small pulmonary nodules, right pleural effusion, bilateral pleural thickening, and right pleural adhesions (**Figure 2D**). Further imaging included a whole-body bone scan, which detected metabolic abnormalities in the T8 and L3 vertebrae. A spinal MRI confirmed metastases in the thoracic 8^th^ and lumbar 3^rd^ vertebrae, with involvement of adjacent bony structures. Ultrasound examination revealed abnormal lymph node enlargement in the bilateral supraclavicular and left subclavian regions. On November 2, 2021, the patient was put on a chemotherapy regimen consisting of bevacizumab combined with oxaliplatin and TAS-102.

## Discussion

Cetuximab is a targeted monoclonal antibody therapy designed to bind to the EGFR specifically (9). By competitively inhibiting EGFR activation, cetuximab effectively blocks signal transduction pathways mediated by endogenous ligands, resulting in the suppression of tumor cell proliferation, inhibition of tumor angiogenesis and metastasis, and induction of apoptosis in malignant cells (10). Its intravenous administration ensures rapid systemic action, making it a critical component of therapeutic strategies for cancers overexpressing EGFR, particularly metastatic CRC.

Clinical evidence demonstrates that cetuximab, when combined with the FOLFIRI chemotherapy regimen, exerts synergistic effects, controlling disease progression and enhancing immune response. This combination therapy has been shown to stabilize biomarkers, improve physical function, and provide superior disease management outcomes. Moreover, cetuximab’s inhibition of DNA topoisomerase I has been implicated in the disruption of DNA replication in tumor cells, further contributing to its antitumor efficacy. However, the therapeutic benefits of cetuximab are often accompanied by significant adverse effects, including dermatologic toxicity, fatigue, gastrointestinal disturbances, and, in rare cases, ILD, which can limit patient compliance and tolerance. The precise mechanisms underlying cetuximab-induced ILD remain unclear, with no unified consensus in the current literature. However, clinical trials and case studies suggest that the onset of cetuximab-related ILD is highly individualized, often influenced by patient-specific factors such as age, baseline pulmonary health, and treatment history. Early-onset ILD has been reported in elderly patients, while delayed onset may occur in others. Patients with pre-existing pulmonary conditions, such as radiation pneumonitis, pulmonary fibrosis, or lung metastases, are at an increased risk of developing ILD during cetuximab treatment.

In our case report, we found an interesting temporal consistency between worsening hypoalbuminemia and ILD progression, which suggest a certain degree of correlation between the two variables. Hypoalbuminemia may promote the occurrence of cetuximab-induced interstitial pneumonia through multiple mechanism. given the high protein-binding property of cetuximab, hypoalbuminemia may increase the concentration of free drug, thereby enhancing its toxicity. Existing studies have demonstrated that hypoalbuminemia is a risk factor for more severe adverse events in patients with solid tumors treated with high protein-binding oral tyrosine kinase inhibitors (TKIs) (11). This mechanism may similarly apply to cetuximab, potentially increasing the likelihood of interstitial pneumonia. Kang et al. (12) identified hypoalbuminemia as a significant risk factor for pulmonary toxicities in patients undergoing cetuximab therapy. hypoalbuminemia is associated with poor prognosis, including higher 90-day mortality rates and shorter treatment durations (11, 13). In patients receiving cetuximab therapy, hypoalbuminemia may further exacerbate the severity of interstitial pneumonia and worsen the prognosis. Additionally, hypoalbuminemia may influence patient responsiveness to treatments such as glucocorticoids. The literature indicates that during TKI treatment, patients with hypoalbuminemia require closer monitoring (11). Closely monitoring albumin levels during treatment is essential to identify at-risk patients early and mitigate potential complications. Similarly, Okamoto et al. (14) conducted a statistical analysis involving 116 head and neck cancer patients treated with cetuximab. Their study highlighted an increased incidence of ILD among patients with concurrent emphysema, underscoring the role of underlying pulmonary conditions in predisposing patients to cetuximab-induced ILD. These observations align with the current understanding that factors such as advanced age, coexisting respiratory diseases, and compromised baseline pulmonary function contribute to the development and severity of cetuximab-induced ILD. There is scarce evidence in the literature directly investigating the relationship between hypoalbuminemia and cetuximab-induced ILD. Nevertheless, considering the mechanism of action of hypoalbuminemia in other drug-induced lung injuries, it can be hypothesized that it may exert a comparable influence in cetuximab-induced ILD. Additional more research is warranted to elucidate its precise mechanism and clinical relevance, not confined to case reports alone.

Typically, patients with ILD present with non-specific clinical manifestations, including symptoms such as cough, fever, and dyspnea. Lung auscultation may reveal crackling sounds in some cases. Pulmonary function tests often indicate hypoxemia, decreased diffusing capacity, and restrictive ventilatory dysfunction. Lung biopsy findings may demonstrate alveolar hemorrhage, diffuse alveolar damage, pulmonary fibrosis, interstitial pneumonia, or bronchiolitis obliterans organizing pneumonia. Although the incidence of cetuximab-associated ILD is low, untreated cases can progress rapidly, leading to high mortality rates. Currently, no targeted therapies are available for cetuximab-induced ILD. To mitigate risks, patients undergoing cetuximab treatment should undergo early chest X-ray or CT imaging upon the presentation of pulmonary signs or symptoms. After excluding pulmonary embolism, clinicians should maintain a high index of suspicion for ILD. If the condition is mild, cetuximab treatment may continue under close monitoring. For severe cases, discontinuation of molecular targeted therapy and initiation of corticosteroid treatment are recommended to control disease progression and improve prognosis.

In summary, cetuximab-induced ILD is an uncommon complication with non-specific clinical presentations. Effective management requires individualized patient care and careful selection of treatment strategies. Early corticosteroid administration plays a critical role in symptom alleviation and reducing mortality rates. This case underscores several underrecognized aspects that warrant further attention. First, the patient’s unique clinical profile, characterized by a history of both lung cancer (a recognized risk factor for ILD) and colorectal cancer, adds complexity to the differential diagnosis and management. Second, we identified a temporal consistency between worsening hypoalbuminemia and ILD progression which suggest a certain degree of correlation between the two variables, a finding that has not been extensively described in the existing literature. Lastly, the delayed onset of ILD after multiple cycles of cetuximab administration contrasts with earlier reports indicating that ILD typically manifests within the first few doses.

## Conclusion

This case report indicates that treatment with cetuximab may be associated with the development of interstitial lung disease (ILD). The patient developed ILD following treatment with cetuximab, despite not having overt respiratory symptoms prior to its onset. This finding underscores the necessity for close monitoring of patients’ pulmonary conditions during clinical use of cetuximab, especially in those with underlying pulmonary diseases. Moreover, this case highlights the importance of monitoring serum albumin levels during treatment, as they may correlate with the severity and progression of ILD. Based on this case, we recommend regular pulmonary function tests and imaging examinations during cetuximab treatment to promptly detect potential ILD. Additionally, for patients presenting symptoms of ILD, it is advisable to consider adjusting the treatment plan, including the possible use of corticosteroid therapy. However, given that this study is a case report, the generalizability of its conclusions is restricted. Further research is warranted to verify the association between cetuximab and ILD and to develop specific treatment guidelines. This case report provides clinical evidence that cetuximab may lead to interstitial lung disease, emphasizing the importance of monitoring and preventing pulmonary complications during the treatment process.

## Data Availability

The original contributions presented in the study are included in the article/supplementary material. Further inquiries can be directed to the corresponding authors.
